# Fasting differentially alters the hypothalamic proteome of chickens from lines with the propensity to be anorexic or obese

**DOI:** 10.1038/s41387-019-0081-1

**Published:** 2019-04-01

**Authors:** Lingbin Liu, Jiaqing Yi, W. Keith Ray, Lucas T. Vu, Richard F. Helm, Paul B. Siegel, Mark A. Cline, Elizabeth R. Gilbert

**Affiliations:** 1 College of Animal Science and Technology, Southwest University, Chongqing, P.R. China; 2 Virginia Tech, Department of Animal and Poultry Sciences, Blacksburg, VA USA; 3 Virginia Tech, Department of Biochemistry, Blacksburg, VA USA; 4 Virginia Tech, Department of Chemical Engineering, Blacksburg, VA USA

## Abstract

**Background:**

The hypothalamus is the ultimate modulator of appetite and energy balance and therefore sensitive to changes in nutritional state. Chicks from lines selected for low (LWS) and high (HWS) body weight are hypophagic and compulsive eaters, respectively, and differ in their propensity to become obese and in their hypothalamic mRNA response to fasting.

**Methods:**

As fasting-induced changes in hypothalamic proteins are unknown, we investigated the hypothalamic proteomes of 5-day old LWS and HWS chicks in the fed and fasted states using a label-free liquid chromatography-tandem mass spectrometry (LC-MS/MS) approach.

**Results:**

A total of 744 proteins were identified in the chicken hypothalamus, and 268 differentially abundant proteins were identified among four pairwise comparisons. Ninety-five proteins were associated with the response to fasting in HWS chicks, and 23 proteins were associated with the response to fasting in LWS chicks. Fasting-responsive proteins in HWS chicks were significantly enriched in ATP metabolic processes, glyoxylate/dicarboxylate metabolism, and ribosome function. There was no enrichment for any pathways in LWS chicks in response to fasting. In the fasted and fed states, 159 and 119 proteins differed between HWS and LWS, respectively. Oxidative phosphorylation, citric acid cycle, and carbon metabolism were the main pathways associated with differences between the two lines of chicks. Enzymes associated with metabolic pathways differed between HWS and LWS in both nutritional states, including fumarase, aspartate aminotransferase, mitochondrial GOT2, 3-hydroxyisobutyrate dehydrogenase, chondrogenesis associated lipocalin, sialic acid synthase, arylamine N-acetyltransferase, pineal gland isozyme NAT-3, and succinate dehydrogenase [ubiquinone] flavoprotein subunit, mitochondrial.

**Conclusions:**

These results provide insights into the hypothalamic metabolic pathways that are affected by nutritional status and the regulation of appetite and eating behavior.

## Introduction

The hypothalamus plays a crucial role in appetite regulation by integrating, coordinating, and transferring multiple nutrient-related signals from both peripheral and central nervous systems^1,2^. The arcuate nucleus (ARC), ventromedial hypothalamic nucleus, dorsomedial nucleus, paraventricular hypothalamic nucleus, suprachiasmatic nucleus, and lateral hypothalamus constitute the “core” of the appetite regulatory network in the hypothalamus. Previous studies have shown that fasting induces changes in glucose and lipid metabolism and gene expression of appetite regulatory peptides in the mammalian hypothalamus^3–6^. However, relevant regulatory mechanisms involved in the response to fasting in the hypothalamus are complex and remain unclear, especially in non-mammalian species.

Proteomic studies can reveal the complex and dynamic biological processes that operate during development and growth, as well as aid in determining environmental effects. Mass spectrometry-based proteomics techniques facilitate the characterization of the protein profile of a sample^7^, via qualitative and quantitative measurements, and assessments of posttranslational modifications and interaction of proteins^8^. To date, proteomics has been applied in chickens principally by studies involving embryos, muscle, adipose tissue, liver, spleen, egg and bursa of Fabricius^9–13^.

Through long-term continuous selection (58 generations) for low (LWS) or high (HWS) juvenile body weight, the Virginia lines of chickens have a more than 14-fold difference in body weight at 56 days post-hatch^14^. The HWS line individuals are all hyperphagic and obese as juveniles whereas the LWS line is comprised of lean individuals with different severities of anorexia^14–16^. The oftentimes severe anorexic condition in LWS chicks manifests itself at an early age as a portion of chicks from this line do not eat and die within the first week post-hatch following yolk sac resorption^17^. As the LWS become obese after lesioning of the ventromedial hypothalamus^18^ and the feeding responses of many appetite-associated neuropeptides are different between the lines, it is hypothesized that the differences in appetite regulation predominantly reside in the hypothalamus. The threshold in food intake responses to anorexigenic factors including amylin, α-melanocyte-stimulating hormone (MSH), corticotropin-releasing factor, ghrelin, insulin, and neuropeptide AF are significantly lower in LWS than HWS^19^. On the other hand, HWS chicks have a lower threshold in their response to calcitonin, calcitonin gene-related peptide, and neuropeptide S relative to LWS^19^. While several studies have revealed differences in hypothalamic mRNA abundance profiles of appetite-associated factors between LWS and HWS chicks^20–24^, data at the protein level are unavailable. Label-free liquid chromatography-tandem mass spectrometry (LC-MS/MS) combines chromatographic techniques with MS to enhance separation in complex biological mixtures, and has widespread use for relative protein quantification^25^. Here we investigate the response to fasting by comparing the hypothalamic proteomes of LWS and HWS chicks in the fed and fasted states on day 5 post-hatch using a label-free LC-MS/MS proteomic quantification approach.

## Materials and methods

### Animals and tissue sampling

Animal protocols received approval by the Institutional Animal Care and Use Committee at Virginia Tech. The lines of chickens used in this research are generated from a long-term selection experiment for low or high body weight at 8 weeks of age^26^. Details of the selection program have been reviewed^17,27^. The founder population consisted of crosses of 7 inbred lines with the LWS and HWS lines maintained as closed populations. Eggs from age contemporary parents from generation S58 parental stocks were incubated in the same machine. Upon hatching, chicks were caged as a group for the first day and then caged individually in a room at 32 ± 1 °C and 50 ± 5% relative humidity. Chicks were given ad libitum access to a mash diet (21.5% crude protein and 3000 kcal metabolizable energy/kg) and tap water.

Five-day-old male chicks were divided into four groups (*n* = 6 for each group; total 24 chicks): LWS Fed (LFe), LWS Fasted (LFa), HWS Fed (HFe) and HWS Fasted (HFa). Within line, chicks were randomly assigned to the fed or fasted group. The numbers of chicks to be used were derived from a power analysis conducted in JMP^®^ (SAS Inst.) using preliminary data for gene expression treatment means and expected standard deviations, with 90% power, and an alpha level of significance of 0.05. The fasting duration was 180 min as described previously^28–30^, with drinking water provided. Each chick was anesthetized using sodium pentobarbital, and its brain removed. The whole brain was snap frozen upside-down in liquid nitrogen to where the most ventral aspect of the optic lobe was level with the surface of the liquid nitrogen, for 11 s. Cuts were made visually according to the following landmarks: perpendicular to the midline suture a cut was made at the septopallio-mesencephalic tract and at the third cranial nerves. Next, 2.0 mm parallel to the midline two cuts were made and the dorsal cut was made from the anterior commissure to 1.0 mm ventral to the posterior commissure^31^. This block (comprised primarily of the hypothalamus) was immediately wrapped with sterile foil and stored at −80 °C until protein isolation. For processing, samples were identified by a number such that investigators were blind to the line and treatment associated with the sample.

### Protein extraction and LC-MS/MS analysis

All solvents and reagents were either LC/MS or proteomics grade unless noted otherwise. The isolated tissue was homogenized using 5 mm stainless steel beads (Qiagen, MD, USA) and lysis buffer (20 mM Tris-HCl, pH 6.8, 137 mM NaCl, 10% glycerol, and 2 mM EDTA) containing Halt Protease Inhibitor Cocktail (Thermo Scientific), and a Tissue Lyser II (Qiagen). After homogenization, samples were incubated in a rotating shaker at 4 °C for 2 h. Samples were then centrifuged for 20 min at 12,000×*g* at 4 °C and supernatant (1 mL) transferred to a fresh tube. The protein concentration was determined with a BCA Protein Assay Kit (Pierce). Approximately 250 μg protein from each sample was precipitated by the addition of 1 mL methanol (Spectrum Chemicals, New Brunswick, NJ) and incubation at −80 °C for 2 h. Precipitated protein was collected at the bottom of each tube by centrifugation for 20 min at 12,000×*g*. Each pellet was washed once with 1 mL methanol. Protein pellets were resuspended in 250 μL freshly-prepared 8 M urea in 100 mM ammonium bicarbonate (AmBic). Proteins were denatured and disulfides reduced by incubation for 1 h at 37 °C after the addition of 4.5 mM dithiothreitol (DTT) in 100 mM AmBic. Free sulfhydryls were then alkylated by incubation for 30 min at room temperature in the dark after the addition of freshly-prepared 10 mM iodoacetamide (IA) in 100 mM AmBic. Unreacted IA was then neutralized by the addition of 10 mM DTT in 100 mM AmBic and the urea content diluted to 4 M by bringing the final volume to 500 μL by the addition of 100 mM AmBic. Endoproteinase LysC (Wako, Richmond, VA) was added at 1:100 (w/w) and digestions incubated with shaking at 37 °C overnight. The following day urea content was further diluted to 1.4 M by bringing the final volume to 1.43 mL by the addition of 100 mM AmBic and trypsin was then added at 1:50 (trypsin:sample, w/w) and the digestions incubated for four h with shaking at 37 °C.

Peptide digests were desalted using solid phase extraction (SPE) after acidification by the addition of 0.2% (v/v) trifluoroacetic acid (TFA) and 1% (v/v) formic acid. The SPE utilized Sep-Pak® Vac 1cc/50 mg C18 cartridges (Waters Corporation, Milford, MA). Cartridges were conditioned and equilibrated using 1 mL methanol followed by 1 mL solvent Y (98:2 water: acetonitrile containing 0.1% TFA). The sample was then applied to the cartridge followed by 3 × 1 mL washes of solvent Y and the desalted peptides were recovered using 1 mL solvent Z (50:50 water: acetonitrile containing 0.1% TFA). Peptide samples were concentrated to dryness using a centrifugal vacuum concentrator, resolubilized in 250 µL solvent Y by sonication and stored at −20 °C.

The resolubilized peptide samples (3 µg) were analyzed by LC-MS using an Acquity I-class UPLC system interfaced with a Synapt G2-S mass spectrometer (Waters Corporation, Milford, MA). The mobile phases were solvent A (0.1% v/v formic acid; Sigma-Aldrich Corporation, St. Louis, MO) in water (Spectrum Chemicals and Laboratory Products, New Brunswick, NJ) and solvent B (0.1% v/v formic acid; Sigma-Aldrich Corporation, St. Louis, MO) in acetonitrile (Spectrum Chemicals and Laboratory Products, New Brunswick, NJ). The separation was performed using a CSH130 C18 1.7 µm, 1.0 × 150 mm column (Waters Corporation, Milford, MA) at 50 µL/min using a 110 min gradient from 3–40% solvent B. The column temperature was maintained at 45 °C.

Column effluent was analyzed using an HDMS^E^ acquisition method (high-definition mass spectrometry with alternating scans utilizing low and elevated collision energies combined with ion mobility) in continuum positive ion “resolution” MS mode. The following source conditions were used: capillary voltage, 3.0 kV; source temperature, 120 °C; sampling cone, 40 V; desolvation temperature, 350 °C; cone gas flow, 50 L/h; desolvation gas flow, 500 L/h; nebulizer gas flow, 6 bar. Low energy (4 and 2 V in the trap and transfer region, respectively) and elevated energy (4 V in the trap and ramped from 20 to 50 V in the transfer region) scans were 1.2 s each with a *m/z* range of 50 to 1800. The ion mobility separation and transfer wave velocities were 600 and 1200 m/s, respectively. Within the ion mobility cell the wave height was ramped from 10 to 40 V^32^.

A 1.2 s low energy scan was acquired every 30 s of a 100 fmol/µL [Glu1]-fibrinopeptide B (Waters Corporation, Milford, MA) solution (50:50 acetonitrile: water with 0.1 % formic acid) for lock-mass correction. This solution infused at 10 µL/min was introduced into the mass spectrometer through a different source which was also maintained at a capillary voltage of 3.0 kV. These data were collected but not applied until the data were processed.

### Protein identification and label-free quantification

Mass spectrometric data from the first 100 min of the 110-min gradient for each chromatographic run were handled utilizing ProteinLynx Global Server v. 3.0.2 (PLGS, Waters Corporation, Milford, MA). Average chromatographic and mass spectrometric peak width resolutions were set to 0.3 min and 30,000 FWHM, respectively. Mass values were lock-mass corrected based on the *m/z* value of the +2 charge state of [Glu1]-fibrinopeptide B (785.842). Peaks were defined based on low energy, elevated energy and bin intensity thresholds of 250, 25 and 2000 counts, respectively. The final peak list for each sample was then searched against a protein database containing the complete *G. gallus* proteome including isoforms downloaded from Uniprot (www.uniprot.org) on 9/2/2015 and 3 randomized decoy entries for each real entry appended using PLGS. Workflow parameters for the protein identification searches were as follows: 2 possible missed cleavages utilizing Lys-C and trypsin as the protease combination, a fixed modification of carbamidomethylation of cysteine, possible modifications of Gln to pyroGlu when Gln is present at the N-terminus of a peptide and oxidation of Met. The peptide and peptide fragment mass tolerances were 24 ppm. Protein identification searches using PLGS had a false discovery rate of no more than 3%.

Following analysis using PLGS, the results were imported into IsoQuant for further processing^33^. Only those proteins identified by two or more unique peptides were included in the final data analysis. The protein false discovery rate was limited to less than 1%. Peptides containing variable modifications and those with missed cleavages were used for protein quantitation in addition to those that were unmodified and trypsin-specific. The same three peptides were used for quantitation of each protein across all replicates after abundances were normalized to ppm. Proteins with fold changes greater than 1.1 or less than 1/1.1 and *P* < 0.05 were considered differentially abundant. The experimental unit was defined as a single bird and, as such, the values obtained for the two technical replicates were averaged for each bird prior to statistical analysis. Prior to analysis, data distributions were assessed using the Proc Univariate procedure of SAS (SAS Inst., Cary, NC). For the statistical analysis, using Perseus^34^, the model include the main effects of line (LWS and HWS), nutritional state (fed and fasted), and the interaction between them, and post-hoc t-tests were used for pairwise comparisons. The alpha-level of significance was *P* < 0.05.

### Functional enrichment analysis

All proteins identified within chicken hypothalamus were searched using RPS-BLAST against the euKaryotic Orthologous Groups (KOG) database and the proteins’ functions were assigned based on KOG searches. The differentially abundant proteins were annotated using GO (Gene Ontology) and KEGG (Kyoto Encyclopedia of Genes and Genomes) databases in order to best identify the function of each protein. The results of GO terms with *P*-values < 10^−5^ were considered to be significantly enriched, and KEGG pathways with *P*-values < 10^−3^ were considered to be significantly enriched.

## Results

### Identification of proteins in chicken hypothalamus

**Fig. 1:**
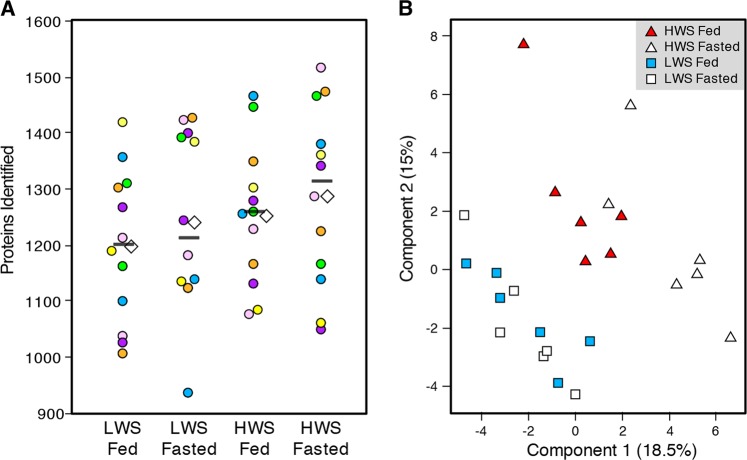
Overview of proteomic analysis of the chicken hypothalamus. **a** The number of proteins identified in each of the four groups. Each colored circle (yellow, blue, orange, green, purple, or pink) is a biological replicate with two per experimental unit (individual chick), with the two representing injection replicates. The white diamond is the average of all runs for that condition and the black bar is the median. *LWS* low body weight-selected line, *HWS* high body weight-selected line, *Fed* ad libitum feeding conditions, *Fasted* fasted for 3 h prior to sample collection. **b** Principal component analysis (PCA) of all samples (each square or triangle is an individual bird). White triangle: HWS Fasted group, red triangle: HWS Fed group, white square: LWS Fasted group, and blue square: LWS Fed group

**Fig. 2:**
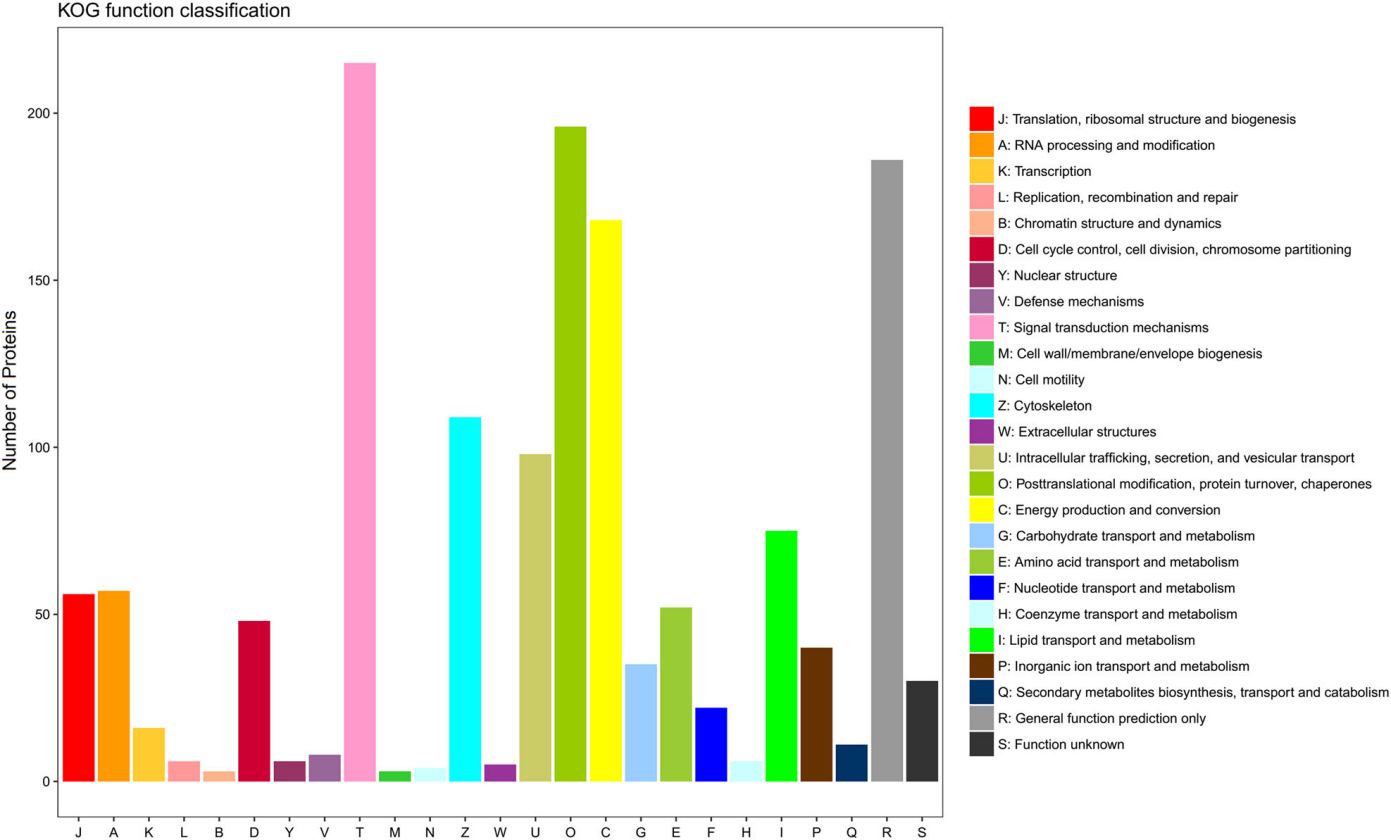
Annotation of the identified proteins as classified by the euKaryotic Orthologous Groups (KOG) categories. The legend corresponds to each category label defined for the bars on the x-axis and the value for each bar represents the number of proteins identified within that category

The HWS chicks weighed more than twice the LWS chicks at the time of sample collection (*P* < 0.0001; 65.3 g ± 2.6 vs. 27.2 g ± 1.1, respectively). Food intake was not recorded during this experiment, however; previous publications document differences in appetite regulation and feeding behavior between the lines^35,36^. The number of proteins identified in each group is shown in Fig. 1a, with averages of 1196, 1240, 1251 and 1286 per biological replicate in LFe, LFa, HFe and HFa groups, respectively. In the 24 libraries, a total of 744 proteins with FDR < 0.01 were identified in the hypothalamus (Table S1). High correlations among all samples were detected, with the Pearson’s correlation coefficients being greater than 0.95 even when comparing different lines or different treatments (Table S2). Principal component analysis (PCA) was used to compare the four separate groups. While the HWS and LWS lines of chicks clearly separated from one another, the effects of fasting were not as clear (Fig. 1b). Functional enrichment using the KOG database revealed clusters associated with signal transduction mechanisms (28.9% of all proteins identified), posttranslational modification, protein turnover, chaperones (26.3%), general function prediction only (25%), and energy production/conversion (22.6%) (Fig. 2).

### Identification of differentially abundant proteins

**Fig. 3:**
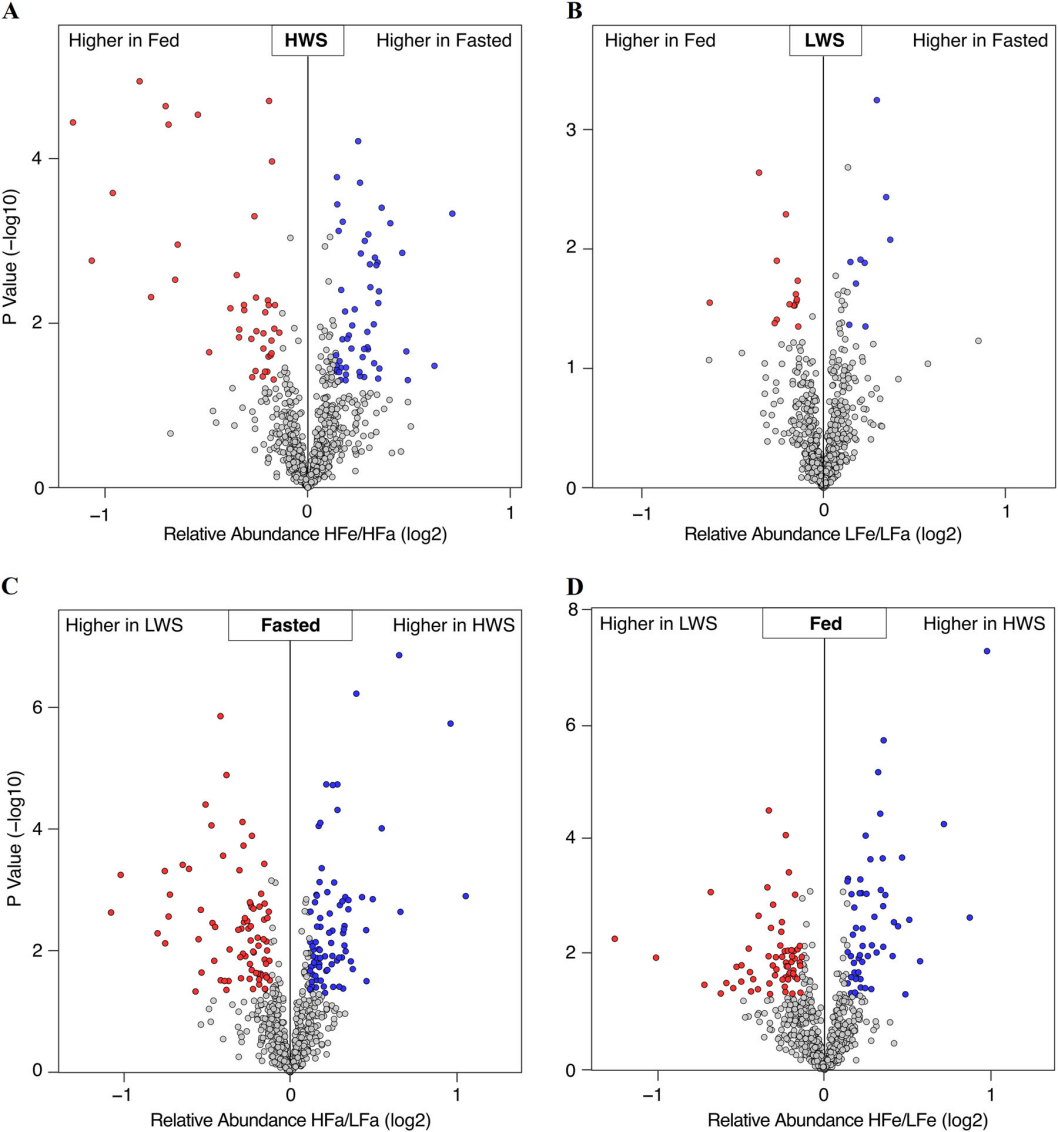
Volcano plots of proteins that were differentially abundant among the four groups. **a** high body weight-selected line (HWS) Fasted (3 h fast) vs HWS Fed (ad libitum conditions); **b** low body weight-selected line (LWS) Fasted vs LWS Fed; **c** HWS Fasted vs LWS Fasted; **d** HWS Fed vs LWS Fed. Red dots represent less abundant proteins, blue dots represent more abundant proteins, and gray dots represent no change

**Fig. 4:**
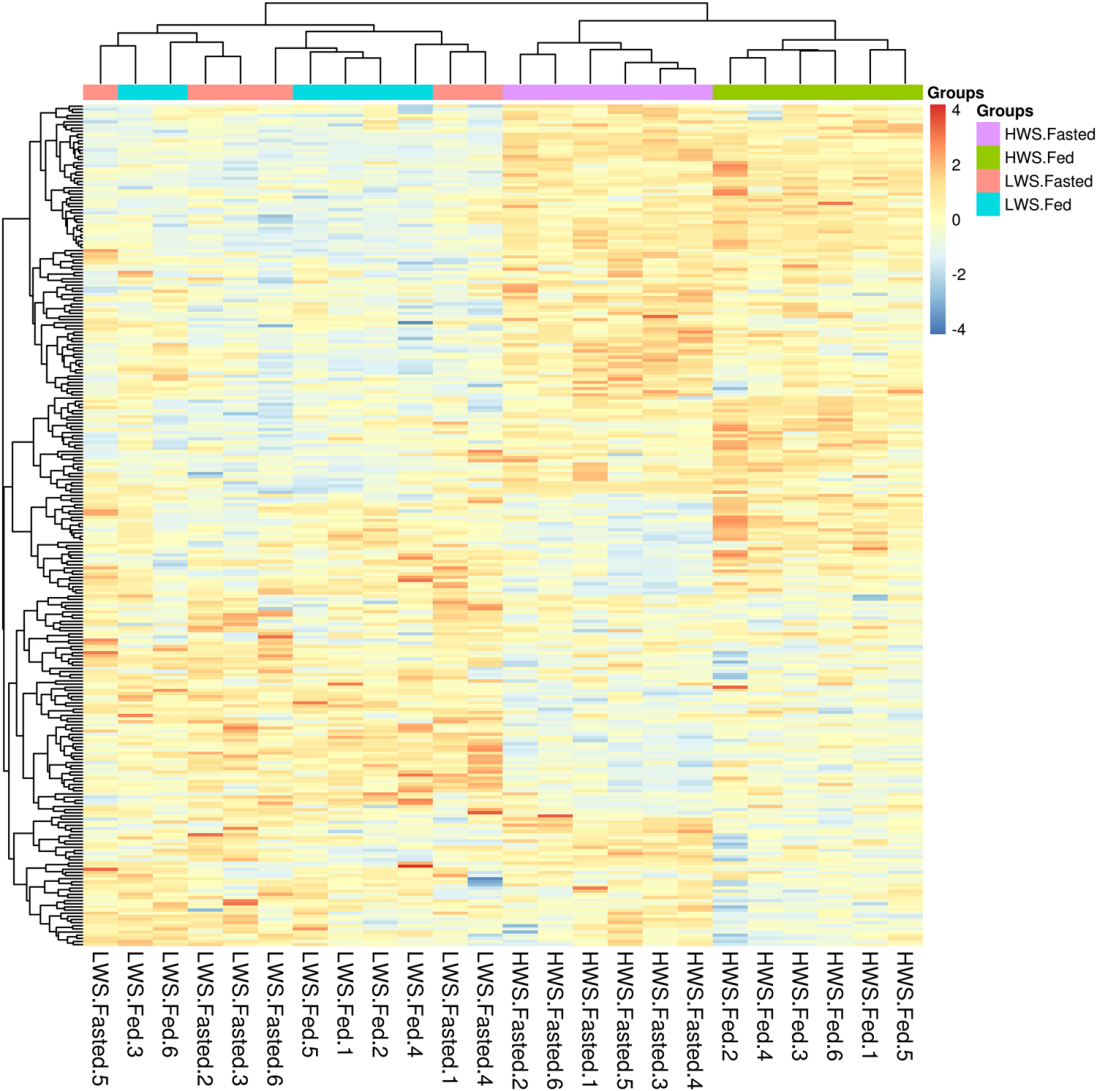
Hierarchical clustering analysis of proteins from 24 samples with 268 differentially abundant proteins. Navy blue, relatively less abundant; firebrick, relatively more abundant. *LWS* low body weight-selected line, *HWS* high body weight-selected line, *Fed* ad libitum feeding conditions, *Fasted* fasted for 3 h prior to sample collection

A total of 95 proteins were differentially abundant (DA) in the fasted and fed states in HWS including 54 that were more abundant (56.8%) and 41 that were less abundant (43.2%) in the fasted state (Fig. 3a). There were 23 proteins that were DA in the fasted and fed states in LWS chicks including 9 that were more abundant (39.1%) and 14 that were less abundant (60.9%) in the fasted state (Fig. 3b). There were 159 proteins that were different between HWS and LWS in the fasted state including 80 that were more abundant (50.3%) and 79 that were less abundant in HWS compared to LWS chicks (49.7%) (Fig. 3c). There were 119 proteins that differed between HWS and LWS in the fed state including 55 that were more abundant (46.2%) and 64 that were less abundant (53.8%) in HWS than LWS (Fig. 3d) (Tables S3-6). Analysis across all relevant comparisons (excluding HFa v. LFe and HFe to LFa) yielded 268 DA hypothalamic proteins (Table S7). To further explore the similarities and relationships within the different libraries, a systematic cluster analysis was carried out to investigate the patterns of DA proteins (Fig. 4). Compared with the six biological replicates for HFa and HFe treatments clustering respectively together, those for LFa and LFe treatments fell into only one subcluster. The libraries of each chicken line also formed a separate cluster.

Among these DA proteins, some were involved in the regulation of neuronal structure including F-actin-capping protein subunit alpha-2 (CAPZA2), contactin-1 (CNTN1), haloacid dehalogenase-like hydrolase domain-containing protein 2 (HDHD2), neuronal growth regulator 1 (NEGR1), septin 2 (SEPT2), and septin 7 (SEPT7). CAPZA2 was less abundant in fasted chicks than fed for both lines (*P* *<* 0.05). CNTN1, NEGR1and SEPT7 increased and HDHD2 decreased in LWS fasted chicks compared to fed. In HWS chicks, SEPT2 was greater in the fasted state than fed (*P* *<* 0.05).

### Function analysis of DA proteins

We carried out GO and KEGG enrichment analyses to identify biological functions of DA proteins for each of the four pairwise comparison groups. The significantly enriched GO terms of the DA proteins between HFa and HFe were mainly related to the biological processes including ATP metabolic processes, peptide metabolic processes, and purine ribonucleoside triphosphate metabolic processes, whereas the molecular functions mostly referred to structural constituents of ribosome, structural molecule activity, and rRNA binding. The result of the KEGG analysis revealed that the significantly enriched pathways were involved in ribosome, and glyoxylate and dicarboxylate metabolism (Table S8). We did not detect any significantly changed GO terms or pathways associated with the proteins that differed between LFa and LFe.

In the HFa and LFa pairwise comparison, the DA proteins were significantly associated with GO terms of molecular function, including GTP binding, purine ribonucleoside binding, and GTPase activity, and oxidative phosphorylation (Table S8). In the HFe and LFe pairwise comparison, the GO term of oxidation-reduction process and KEGG pathways involving the TCA cycle, oxidative phosphorylation, and carbon metabolism were significantly overrepresented (Table S8).

### The relationship between the two lines in response to fasting

**Fig. 5:**
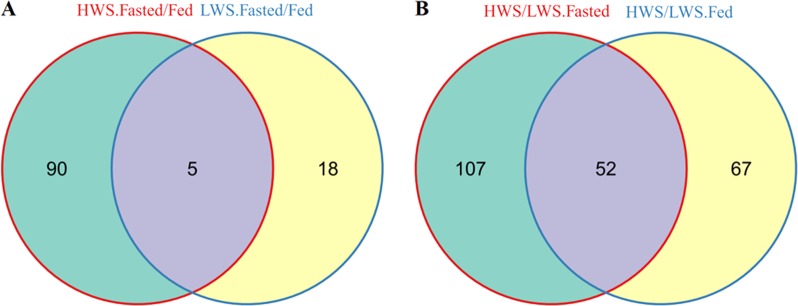
Venn diagram showing the relationship for the differentially abundant proteins among the pairwise comparisons. Numbers represent the number of differentially abundant proteins within the shaded portion. **a** HWS (Fasted/Fed) vs LWS (Fasted/Fed); **b** (HWS/LWS) Fasted vs (HWS/LWS) Fed. *LWS* low body weight-selected line, *HWS* high body weight-selected line, *Fed* ad libitum feeding conditions, *Fasted* fasted for 3 h prior to sample collection

**Fig. 6:**
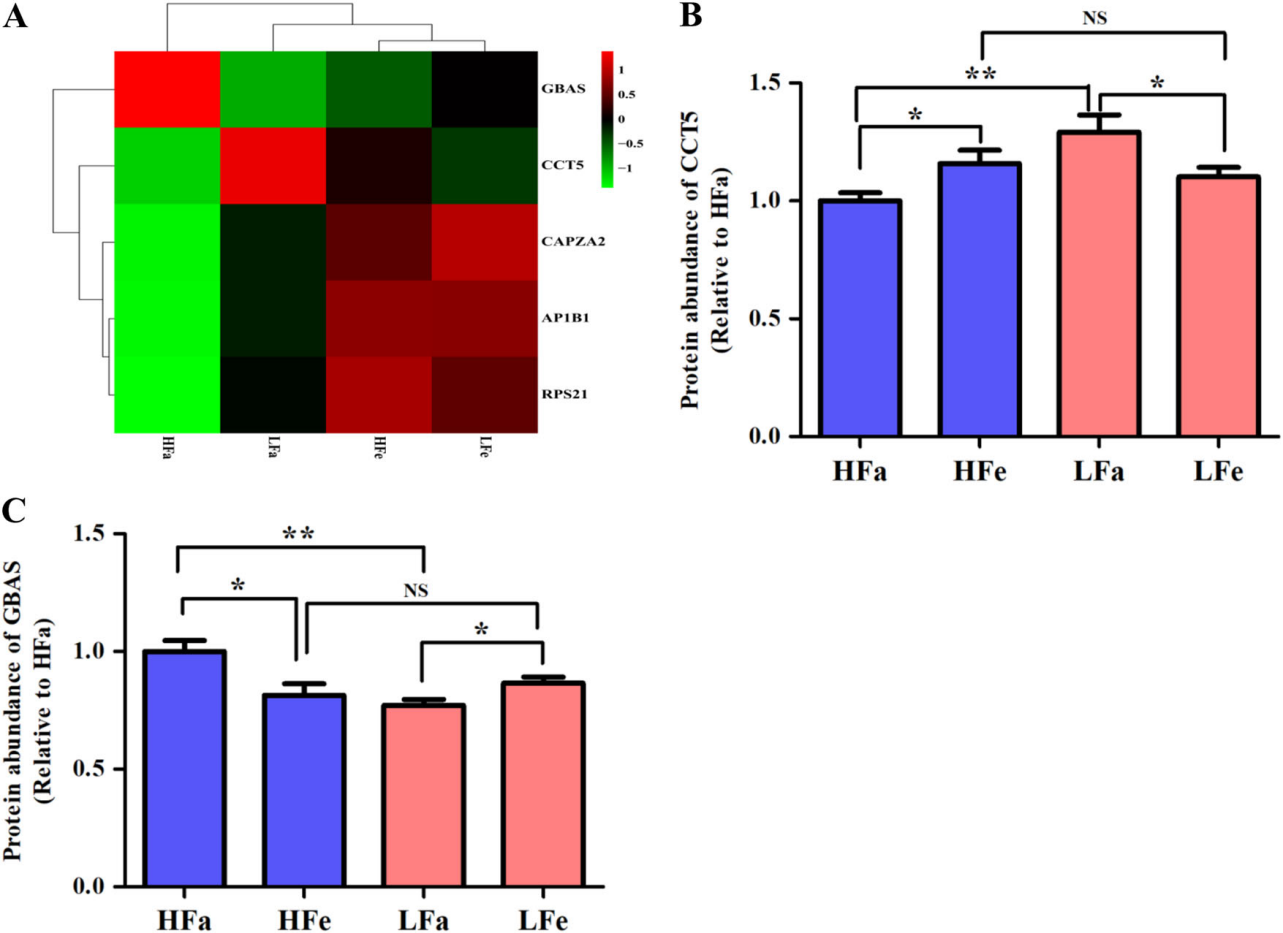
The abundance of five fasting-responsive proteins that overlapped between line chicks. **a** Hierarchical clustering analysis of the five proteins, **b** protein abundance of CCT5 in the four treatment groups, and **c** protein abundance of GBAS in the four treatment groups. AP1B1, adaptor related protein complex 1 beta 1 subunit; CAPZA2, F-actin-capping protein subunit alpha-2; CCT5, chaperonin containing TCP1 subunit 5; GBAS, glioblastoma amplified sequence; RPS21, 40 S ribosomal protein S21. *HFa* high body weight-selected line fasted group, *HFe* high body weight-selected line fed group, *LFa* low body weight-selected line fasted group, and *LFe* low body weight-selected line fed group. All data are presented as least squares means ± SEM. *P *<* 0.05; **P *<* 0.01; *NS* not significant

To further explore the relationship between the two lines in response to fasting, we conducted an interactions analysis of the proteins that were DA. Identified were five fasting-responsive proteins that overlapped between the lines (Fig. 5a), including adaptor related protein complex 1 beta 1 subunit (AP1B1), CAPZA2, chaperonin containing TCP1 subunit 5 (CCT5), glioblastoma amplified sequence (GBAS), and 40 S ribosomal protein S21 (RPS21) (Table 1). Among these, AP1B1, CAPZA2, and RPS21 displayed the same pattern (Fig. 6a), and all were significantly less abundant in response to fasting in both lines (*P* *<* 0.05). In addition, CCT5 was less abundant in HWS chicks after fasting (*P* *<* 0.05), and more abundant in LWS chicks in response to fasting (*P* *<* 0.05) (Fig. 6b). In contrast, GBAS was more abundant in HWS and less abundant in LWS (*P* *<* 0.05) (Fig. 6c). CCT5 and GBAS also differed between the two lines in the fasted state (*P* *<* 0.01), whereas there was no difference between lines in the fed state (*P* *>* 0.05). Among these five proteins, AP1B1, CAPZA2, RPS21 and CCT5 were positively correlated (*P* *<* 0.05) (Table S9).

**Table 1:** Five fasting-responsive proteins that overlapped between LWS and HWS chicks

Accession	*P*-value (HFa vs HFe)	HFa/HFe	*P*-value (LFa vs LFe)	LFa/LFe	Description
A0M8U0	0.00003	0.62	0.00	0.78	F-actin-capping protein subunit alpha-2, CAPZA2
E1BSJ2	0.00002	0.61	0.03	0.9	40 S ribosomal protein S21, RPS21
F1NCI1	0.00002	0.69	0.01	0.87	adaptor related protein complex 1 beta 1 subunit, AP1B1
F1NZ68	0.02	1.23	0.03	0.89	glioblastoma amplified sequence, GBAS
Q5F411	0.04	0.86	0.04	1.17	chaperonin containing TCP1 subunit 5, CCT5

*HFa/HFe* protein abundance in HWS fasted chicks/protein abundance in HWS fed chicks, *LFa/LFe* protein abundance in LWS fasted chicks/protein abundance in LWS fed chicks.

### Differences between LWS and HWS in hypothalamic proteins

We also detected 52 DA proteins that overlapped between HWS and LWS in both nutritional states (Fig. 5b and Table S10). Among these proteins, there were some enzymes that were associated with metabolic pathways, including fumarase (FH), aspartate aminotransferase, mitochondrial (GOT2), 3-hydroxyisobutyrate dehydrogenase (HIBADH), chondrogenesis associated lipocalin (L-PGDS), sialic acid synthase (NANS), arylamine N-acetyltransferase, pineal gland isozyme NAT-3 (PNAT3) and succinate dehydrogenase [ubiquinone] flavoprotein subunit, mitochondrial (SDHA) (Table 2). The protein abundance of GOT2, PNAT3 and SDHA was greater in LWS than HWS chicks in both fasted and fed states, whereas FH, HIBADH, L-PGDS and NANS were greater in HWS than LWS in both nutritional states. NANS differed by about twofold between the lines.

**Table 2:** Metabolic enzymes and transporters that differed between LWS and HWS chicks

Accession	HFa/LFa	HFe/LFe	Ratio	Description
P00508	0.66	0.85	0.77	Aspartate aminotransferase, mitochondrial, GOT2
P13914	0.89	0.87	1.01	Arylamine N-acetyltransferase, pineal gland isozyme NAT-3, PNAT3
Q5ZLD1	1.26	1.19	1.06	Fumarase, FH
Q5ZLI9	1.16	1.10	1.05	3-hydroxyisobutyrate dehydrogenase, HIBADH
Q5ZMH8	2.21	1.83	1.21	Sialic acid synthase, NANS
Q8QFM7	1.29	1.42	0.91	Chondrogenesis associated lipocalin, L-PGDS
Q9YHT1	0.85	0.87	0.97	Succinate dehydrogenase [ubiquinone] flavoprotein subunit, mitochondrial, SDHA

*HFa/LFa* protein abundance in HWS fasted chicks/protein abundance in LWS fasted chicks, *HFe/LFe* protein abundance in HWS fed chicks/protein abundance in LWS fed chicks. Ratio = (HFa/LFa) / (HFe/LFe)

To further illustrate the regulatory network among overlapping proteins, we conducted a PPI (protein-protein interaction) network analysis using software STRING (http://version10.string-db.org/) (Supplemental Figure). In the present study, we focused on seven factors involved in the metabolic pathways described above in the protein interaction networks. SDHA was identified as a “hub” protein for line differences. GOT2 and FH directly interacted with SDHA, and HIBADH had an indirect interaction with these three proteins.

## Discussion

Proteomics allows for the global, quantitative analysis of dynamic changes in cellular proteins to reveal protein relationships and functions and to identify specific or new proteins^8,37^. At present, there are few proteomics studies related to hypothalamic function in poultry. Using a 2-dimensional gel electrophoresis-based approach, Kuo et al. identified six spots containing proteins that related to regulating gene expression, signal transduction and lipid metabolism using hypothalamic proteins isolated from high and low egg production strains of chickens^38^. In this study, we identified 744 proteins in the chicken hypothalamus using label-free LC-MS/MS. Based on the KOG analysis, the functions of these proteins were mainly related to signal transduction mechanisms, posttranslational modifications, protein turnover, chaperones, and energy metabolism.

We found changes in hypothalamic protein abundances in response to fasting in both LWS and HWS chicks and the high correlations indicated that the hypothalamic proteomes of the HWS and LWS chicks are very similar. This suggests that differences in body weight may then be due to change in posttranslational changes of proteins or differences in the abundance of neuropeptides or receptors that were not captured in the present analysis. The PCA plot analysis of all proteins identified and systematic cluster analysis of DA proteins showed similar results, with tight clustering between LFa and LFe groups. The number of DA proteins in response to fasting in HWS chicks was about four-fold greater than in LWS suggesting that the LWS are possibly less sensitive to fasting.

In this study, 5 fasting-responsive proteins that overlapped between the lines are associated with fatty acid metabolism or proteasome and ribosome function. AP1B1 was implicated in the function of SREBP-1 (sterol regulatory element binding protein 1), which is a critical regulator of fatty acid homeostasis^39^. CAPZA2, a cytoskeleton assembly-associated protein, was differentially expressed in brains of Paraoxonase 1 (Pon1) -null mice^40^. RPS21 is a component of the small 40 S ribosomal subunit^41^. In our study, AP1B1, CAPZA2 and RPS21 displayed the same pattern (elevated in fasted animals) and their protein abundance presented certain significant correlations. These results indicated that those factors had tight associations in response to fasting in the chick hypothalamus, which could indicate coordinated regulation of their genes’ expression. In addition, CCT5 belongs to the Chaperonin Containing T-complex protein-1 (CCT) family that plays an important role in ensuring efficient folding of nascent or stress-denatured proteins^42^. GBAS was defined as a candidate gene for combined deficiencies in the oxidative phosphorylation system (OXPHOS) and its mRNA was most abundant in brain, skeletal muscle and heart^43^. In this study, CCT5 was less abundant in HWS fasted chicks versus HWS fed chicks, but more abundant in fasted LWS chicks than in LWS fed chicks. This observation contrasts with the expression pattern of GBAS, which was more abundant in HWS fasted than fed chicks and in LWS fed than fasted chicks. These results suggest that the two proteins were associated with different responses to fasting in the lines.

It should be noted that the GO term ATP metabolic process as well as the KEGG pathway of glyoxylate and dicarboxylate metabolism were significantly enriched in proteins that differed between HFa and HFe. Glyoxylate and dicarboxylate metabolism is a key link to carbohydrate metabolism, which makes energy available to cells and temporarily stores the released energy in the form of high energy molecules^44^. These results suggest that HWS chicks may stimulate the use of carbohydrates as fuel for metabolic needs. There was also enrichment in purine ribonucleoside triphosphate metabolic process, structural constituent of ribosome, rRNA binding, and ribosome pathways, indicating that fasting altered ribosome function in HWS chicks. Although some proteins changed in response to fasting in LWS chicks, we did not identify any changed GO terms or KEGG pathways, which agrees with the results of the PCA plot analysis and systematic cluster analysis. These results indicate that LWS chicks are less sensitive to the effects of fasting on hypothalamic function.

Several factors that were altered by fasting in both LWS and HWS were structural in nature. Neuronal growth regulator 1 (NEGR1) is a factor that plays a role in cell adhesion and axonal regeneration and may be involved in the control of body weight and food intake^45,46^. Lee et al. (2012) reported that NEGR1 mRNA was found in distinct hypothalamic nuclei and disruption of NEGR1 in mice resulted in a reduction in body weight^45^. In rats, food restriction increased the mRNA expression of NEGR1 in the ARC and VMH^46^, similar to the increased NEGR1 abundance in LWS. Septins assemble into cytoskeletal filaments and are required for development, activity, and connectivity of a functional nervous system^47^. Septins comprise complexes to perform different biological functions in various cell types and at different developmental stages, and SEPT7 is a ubiquitous subunit. The complex SEPT2/7 has been described^47^. Lee et al. showed that when a member of a complex was suppressed (RNA interference or gene knockout), the other member of the complex was also affected^47^.

In our study, SEPT7 was greater in LWS fasted than fed chicks. In HWS chicks, SEPT2 was increased in the fasted state. HDHD2 was recently characterized as containing a phosphatase activity^48^ and is related to hypertension in humans. The HWS fasted chicks had higher levels of the phosphatase ACP1 relative to the fed HWS chicks. These changes support the hypothesis that the phenotypes observed are part of a posttranslational pathway involving control of phosphorylation levels. The alterations of these structural proteins indicate that fasting may induce changes in the fundamental structure of the nervous system in chicks, which may be especially relevant at the young age studied in the present research (5 days post-hatch).

Ka et al.^20–22^ and Zhang et al.^23,24^ reported differences in gene expression profiles in the hypothalamus at the mRNA level in the lines reported in our study. Many differentially expressed genes were identified including some involved in neuronal plasticity and monoamine synthesis/metabolism and neurotransmitter function, respectively. Here we describe hypothalamic proteins in the LWS and HWS lines. Whether in the fed state or fasted state, there were numerous proteins that differed between them. In general, oxidative phosphorylation, TCA cycle, and carbon metabolism were the main pathways associated with line differences. This finding is consistent with several previous observations at the transcriptional level, suggesting that metabolic activities differ in the hypothalamus^24,49–53^.

FH, as a mitochondrial isoenzyme, catalyzes conversion of fumarate to malate in the tricarboxylic acid (TCA) cycle^54^. The enzyme GOT2 plays a key role in amino acid metabolism and the urea and tricarboxylic acid cycles, and produces aspartate (a precursor of N-acetylaspartate) from oxaloacetate and L-glutamate in mitochondria^55^. HIBADH belongs to the family of oxidoreductases, specifically acting on the CH-OH group of donors with NAD+ or NADP+ as acceptors, and participates in leucine, isoleucine and valine degradation^56^. Sialic acid synthase is encoded by the NANS gene and is a key enzyme that functions in the biosynthetic pathways of sialic acids^57^. PNAT3 mainly participates in arylamine N-acetyltransferase activity, and the two substrates are acetyl-CoA and arylamine, and 2 products are CoA and N-acetylarylamine^58,59^. SDHA, as the flavoprotein subunit of succinate dehydrogenase, is involved in complex II of the mitochondrial electron transport chain, and participates in step 1 of the subpathway that synthesizes fumarate from succinate and electron transfer from succinate to ubiquinone in mitochondria^60^. These above-mentioned enzymes are important in metabolic pathways^54–60^. Results of the present study thus suggest that there is greater regulation of energy metabolic pathways in HWS than LWS, which supports that these chicks eat more and in general are in a more positive energy balance than LWS.

NANS-mediated sialic acid synthesis is required for brain and skeletal development. Brain contains the highest concentration of sialic acid among the body organs^61^. Sialic acid is an essential component of sialylated sphingolipids such as brain gangliosides^62^. Genetic deficiency of glycoprotein synthesis leads to infantile epilepsy and developmental arrest, suggesting that appropriately sialylated glycoproteins are necessary for higher brain functions^63^. Neu5Ac (N-acetylneuraminic acid), as the predominant structure of sialic acid, is the key monomeric precursor of polysialic acid glycan, which posttranslationally modifies the cell membrane-associated neural cell adhesion molecules (NCAM)^63^. Sialic acid synthesis has a pivotal role in normal function of several key players in bone and cartilage growth and development, including bone sialoprotein, osteopontin and chondroitin sulfate proteoglycans^64–66^. Intellectual developmental disorders, and brain and skeletal dysplasias were observed in NANS-deficient individuals, suggesting that the requirements for sialic acid in the developing brain and skeletal muscle must be met by endogenous synthesis of sialic acid through the NANS pathway^63^. Interestingly, NANS was about twofold more abundant in HWS than LWS in both nutritional states. The results suggest that the lean and hypophagic phenotype of the LWS chicks could be associated with having less hypothalamic NANS.

## Conclusion

In summary, a total of 744 proteins were identified in the chicken hypothalamus, and 268 differentially abundant proteins were identified among four pairwise comparisons.

There were 95 proteins associated with the response to fasting in HWS chicks, and 23 proteins associated with the response to fasting in LWS chicks. Fasting-responsive proteins in HWS chicks were significantly enriched in ATP metabolic process, glyoxylate and dicarboxylate metabolism, and ribosome function. In contrast, no pathways were enriched in response to fasting in LWS chicks. A total of 159 proteins differed between HWS and LWS in the fasted state, and 119 in the fed state. Oxidative phosphorylation, TCA cycle, and carbon metabolism were the main pathways associated with the difference between the two lines. Seven enzymes associated with metabolic pathways were different between LWS and HWS chicks in both nutritional states, including FH, GOT2, HIBADH, L-PGDS, NANS, PNAT3 and SDHA. The current study is the first to investigate the hypothalamic response to fasting through proteomics in LWS and HWS chicks. Our data identify a subset of proteins that are potentially important regulators of hypothalamic function in chicks in the fasted state, but further studies are necessary for functional validation.

## Supplementary information

Supplemental Figure

Supplemental Tables

## Data Availability

ProteomeXchange Consortium via the PRIDE partner repository with the dataset identifier PXD009086. Project 10.6019/PXD009086. Project Name: Fasting differentially alters the hypothalamic proteome of chickens from lines selected for low or high body weight. FOR REVIEWER PURPOSES: Website: www.ebi.ac.uk/pride/archive/login Username: reviewer16589@ebi.ac.uk; Password: XJwSKn9f
