# Supplementary figures and images for: Fasting differentially alters the hypothalamic proteome of chickens from lines with the propensity to be anorexic or obese
Source: Nutr Diabetes. 2019 Apr 1;9:13. doi: 10.1038/s41387-019-0081-1 (PMC6443654; doi:10.1038/s41387-019-0081-1)

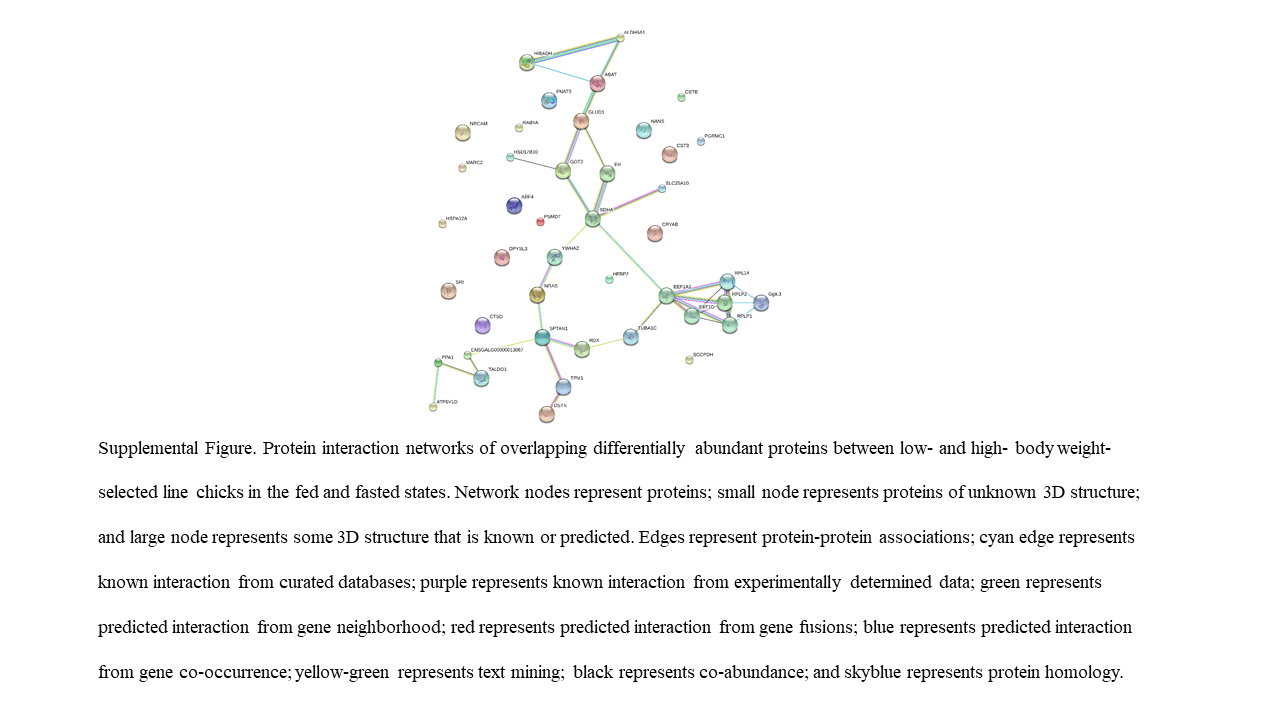

Supplement: Supplementary file 1 — Supplemental Figure [file 41387_2019_81_MOESM1_ESM.tif]
